# Supporting the Return to Work of Breast Cancer Survivors: From a Theoretical to a Clinical Perspective

**DOI:** 10.3390/ijerph19095124

**Published:** 2022-04-22

**Authors:** Bertrand Porro, Mario Campone, Philippe Moreau, Yves Roquelaure

**Affiliations:** 1 Univ. Angers, Univ. Rennes, Inserm, EHESP, IRSET (Institut de Recherche en Santé, Environnement et Travail)—UMR_S 1085, SFR ICAT, F-49000 Angers, France; yves.roquelaure@univ-angers.fr; 2 Oncology Department, Institut de Cancérologie de l’Ouest, F-44805 Saint-Herblain, France; mario.campone@ico.unicancer.fr; 3 Center for Research in Cancerology and Immunology Nantes-Angers, Inserm UMR 1232, Univ. Nantes and Univ. Angers, F-44307 Nantes, France; 4 University Hospital Hôtel-Dieu, 44000 Nantes, France; philippe.moreau@chu-nantes.fr; 5 University of Nantes, 44035 Nantes, France; 6 Univ. Angers, CHU Angers, Univ. Rennes, Inserm, EHESP, IRSET (Institut de Recherche en Santé, Environnement et Travail)—UMR_S 1085, SFR ICAT, F-49000 Angers, France

**Keywords:** breast cancer, survivors, return-to-work, clinical framework

## Abstract

Promoting the return to work of breast cancer survivors is of major interest to patients, healthcare and occupational health professionals, companies, governments, and researchers worldwide. We previously conducted a French consensus study resulting in a model describing the multifactorial process of the return to work of breast cancer survivors (the REWORK-BC model). Other work has identified the transtheoretical model as a relevant theoretical framework for interventions to promote the return to work of cancer survivors. In this opinion paper, we provide a theoretically-based clinical framework describing how to support breast cancer survivors at each stage of the return-to-work process. This clinical framework considers several essential aspects of supportive care for breast cancer survivors returning to work, such as: (i) helping the patient actively self-manage, by considering her to be the main decision-maker; (ii) respecting and adapting to the patient’s choice of professional project; (iii) respecting the temporality of the patient’s choices; (iv) proposing tailored interventions; (v) implementing simple tools to promote the return to work, shared representation between the patient and a multidisciplinary team, and improvement of working conditions and the knowledge of health and occupational professionals, and managers or employers; and (vi) maintaining certain flexibility aimed at proposing, but never imposing, changes in practices. This clinical framework, specific to breast cancer survivors, could be extrapolated to other tumor types, offering a practical guide for healthcare and occupational health professionals to better understand the return-to-work process of cancer survivors. This clinical framework aims to be a usable tool for any hospital or cancer care center wishing to implement a patient-centered intervention that promotes returning to work, regardless of the country.

## 1. Introduction

Over the two past decades, improvements in breast cancer treatments have led to an increase in patient survival [1,2]. For most working-age breast cancer survivors (BCSs), treatments and their long-term side effects can make it difficult to return to work (RTW) [3,4,5]. Scientific investigations have identified several determinants of BCSs’ RTW, and the role of each stakeholder involved in the process, namely the BCS, healthcare and occupational health professionals, and managers/employers [3,6,7]. Healthcare professionals within the hospital, as well as occupational health professionals and managers within the company play an important role in providing advice, support, and functional and emotional assistance to the BCSs in the RTW process [8,9]. However, they highlight a lack of knowledge, skills, and tools to support the sustainable RTW of BCSs [6,10].

Interventions to promote RTW for BCSs must be theory-based and patient-centered [11,12,13]. Duijts et al. identified the Transtheoretical Model of Behavior Change (TTM) as a relevant theoretical framework for developing these interventions [14,15]. A recent qualitative study endorses the value of tailoring support for the work participation of cancer survivors along the stages of change described by the TTM [16]. However, the TTM alone cannot account for all the complexities of behavior change, particularly when studying the multidimensional process of RTW [13,14]. The recently proposed multidimensional REWORK-BC model may complement the TTM by providing a multidisciplinary framework to assess each determinant at the appropriate time, and facilitate supportive care for BCSs’ RTW [13].

## 2. Aims

We here aim to propose a multidimensional framework for managing the RTW of BCSs from both a theoretical and clinical perspective. After presenting the TTM, the REWORK-BC model, and their complementarity, we will suggest the determinants to be assessed at each stage of the RTW process as well as the assessment indicators for each stage of the process will be suggested.

## 3. A Theoretical Perspective

### 3.1. The Transtheoretical Model

The TTM describes the following six stages of change, with varying temporality [14]:

- Stage 1—Precontemplation: no intention to change behavior within the next six months.
- Stage 2—Contemplation: intention to change behavior in the next six months.
- Stage 3—Preparation: intention and plans to act in the near future, (i.e., around one month).
- Stage 4—Action: behavior has changed within the past six months, with a high risk of relapse.
- Stage 5—Maintenance: new behavior undertaken for more than six months and prevention of relapses, which are less frequent.
- Stage 6—Termination: no temptation to relapse and full confidence to maintain the new behavior [14].

The notion of relapse is an integral part of the process and must be well managed and de-dramatized to prevent people from abandoning the change process altogether [14].

In addition, change processes are often omitted in TTM evaluations even though they facilitate the transition from one phase to another and thus constitute the objectives to be progressively achieved during therapeutic support [14]. Empirical studies describe ten change processes involved in the TTM as follows [14,17,18,19]:

- The transition from the precontemplation phase to the contemplation phase depends on consciousness-raising, dramatic relief, and environmental reevaluation that can be enhanced by awareness campaigns, testimonials, (e.g., by peer support), or transmission of information.
- To reach the preparation phase, the patient sets up a process of self-reevaluation which can be enhanced by clarifying her values.
- Self-liberation, indicating that the patient is convinced that she can act.
- Maintenance can be achieved if counterconditioning, (e.g., by increasing assertiveness strategies), helping relationships, reinforcement management, (e.g., through peer recognition and support), and stimulus control, (e.g., by changing the environment) are adopted.
- Social liberation, requiring an increase in social opportunities and alternatives, especially for people who are relatively deprived or oppressed. This process may require several stages of change [14].

According to the TTM, progression through the stages of change and achievement of change processes is based on: (i) perception of the benefits and drawbacks of changing behavior, (i.e., decisional balance); (ii) feeling to be able to progressively achieve the steps necessary for change, (i.e., self-efficacy), which should increase over time; and (iii) intercurrent events that may make it difficult to change [14].

### 3.2. The REWORK-BC Model

The REWORK-BC model emerged from a French expert consensus [13]. This conceptual model offers an integrative, dynamic, and multidisciplinary perception of the RTW of BCSs [13]. Its strength lies in putting individual capacities into perspective with work demand, thus defining the Margins of Maneuver (MM) for the RTW [20,21]. It implements (i) the initial MM corresponding to the work situation experienced before the cancer diagnosis; (ii) the therapeutic MM corresponding to the gain of MM following the implementation of supportive care and/or accommodations in the work environment to facilitate RTW; and (iii) the final MM corresponding to the readjustments of the working situation after RTW, that are necessary for sustainable job retention [13,20,21]. In addition, the REWORK-BC model articulates from a transactional perspective: (i) dispositional variables, (e.g., socio-demographic, socio-professional, financial, medical); (ii) a primary appraisal related to work ability; (iii) a secondary appraisal corresponding to individual and social resources; (iv) individual and professional adjustments strategies; (v) outcomes in terms of RTW or non-RTW; and systematic feedback providing the dynamics of work ability, resource and adjustment strategy re-evaluations [13].

### 3.3. Complementarity between TTM and REWORK-BC Models

The TTM is a generalist model of behavior change that has been widely proven in framing interventions to promote health behaviors in patients diagnosed with cancer [22,23,24,25]. It adds a temporal aspect to the REWORK-BC model, enabling an intervention aimed at promoting the RTW of BCSs to be framed. In turn, the REWORK-BC model is very specific to the RTW of BCSs, providing several typologies of BCSs regularly encountered in clinical practice and the precise elements to be evaluated at each stage of change [13]. It offers a specific clinical view and favored patient-centered interventions [13]. Both models will consider some form of re-evaluation of the situation: (i) the TTM through the consideration of relapses to the previous stage of behavior [14]; and (ii) the REWORK-BC model taking systematic feedback into account [13].

## 4. A Clinical Perspective

We argue that promoting the RTW of BCSs is essentially based on three aspects: (i) enhancing the health-related quality of life [26,27]; (ii) adjusting the work situation, work organization, and management practices [6,28,29]; and (iii) coordinating stakeholders, (i.e., BCSs, healthcare professionals and managers), while considering the specific needs of each patient.

Because of the multiplicity of the stakeholders, and their varying perspectives on the situation, the coordination may be complex. A protocol that is too strict and a course of action that is too standardized might lead to a lack of agility during the RTW process. Indeed, RTW expectations vary according to individual situations, companies, legislation, and insurance systems [13,30,31,32]. Consequently, we argue that RTW support for BCSs should be based on a sufficiently flexible clinical framework and the provision of a range of simple tools. The tools made available must: (i) meet the specific needs of each stakeholder; and (ii) suggest, without imposing, ways of improving practices.

### 4.1. Functionality of Simple Tools

Table 1 shows the different simple tools included in the clinical framework and their functionality at each stage of the RTW process. 

The implementation of the set of tools would build on both the steps and processes of the TTM and the assessment elements of the REWORK-BC model, to facilitate the RTW process of BCSs while considering the readjustment phases, (i.e., feedback, whether there are behavioral relapses or not) [13]. The clinical implications and elements to be assessed at each stage of the process are detailed below.

### 4.2. Stage 1—Precontemplation

At this point, the patient has generally just been diagnosed and is beginning her care journey. The focus is on care, with the main objective being recovery. Consequently, the RTW is not yet envisaged [5,15].

***Promoting the achievement of the contemplation phase*.** The patient’s awareness of the importance of preparing for the RTW at an early stage must be promoted. Posters could be put up in healthcare professionals’ offices which would lead the patient to talk about it as early as possible (Table 1). An informative booklet or leaflet with a website that summarizes, with respect to medical privacy, key information and testimonials from patients, RTW specialists, and managers could also be helpful (Table 1) [33]. The patient would be able to consult these materials, which are available to them, whenever they feel the need. On the website, there could be a page dedicated to managers and employers indicating the main actions to be taken, at each stage of the disease, to support the RTW of the employee diagnosed with breast cancer, (e.g., the Missing Link: optimizing RTW of Employees diagnosed with cancer, by Supporting employers—MiLES intervention) [9,28,29]. Patients could be invited to forward this web page to their supervisors if they so wish.

### 4.3. Stage 2—Contemplation

Breast cancer patients expect to RTW within six months, usually after completion of chemotherapy [5,15]. At this stage, the main contacts are the oncologist, the radiotherapist, the general practitioner, and/or the occupational health physician. Simple information can be collected in the medical file by these professionals (Table 2). Recording the socio-demographic, socio-professional, and financial characteristics will enable them to identify BCSs who need special attention, based on prognostic factors of adverse occupational outcomes, and allow an early contact with a social worker, if necessary, in order to start the administrative and financial procedures, (e.g., financial emergency).

Recording the medical characteristics will enable the potential physical and psychological side effects of treatment affecting the RTW, (e.g., fatigue, pain, emotional distress) to be predicted [34,35]. For those healthcare professionals lacking the time and knowledge to have an in-depth discussion on the RTW [10], one solution would be to propose a referral to an RTW coordinator (e.g., nurse, social worker, psychologist) to support each patient in her RTW process [36,37]. Durand et al. recently identified the main competencies of the RTW coordinators, namely [37]:

- *Competency 1*: Tailoring practices to the needs of the BCSs throughout the RTW process.
- *Competency 2*: Involving the workplace stakeholders as much as possible.
- *Competency 3*: Rethinking/questioning practices and ideas regarding the RTW process and, as much as possible, inviting the stakeholders in the hospital and the workplace to do the same.
- *Competency 4*: Developing practices that comply with laws, regulations, agreements, and specific procedures related to the BCSs’ RTW process.

***Promoting the achievement of the preparation phase: assess and clarify values (Table 3)*.** The main objective of the first interview with the RTW coordinator is to assess and clarify the patients’ values regarding their work. The goal is to better understand the patient’s professional project and to discuss the financial consequences of their decision. In our clinical practice, we have identified several types of career plans:

- Returning to previous work. An initial assessment of the RTW self-efficacy may be relevant in order to establish a basal score and better understand the feelings surrounding RTW [38].
- Disability pension, or early retirement for the oldest patients [39]. Contact with a social insurance physician, a social worker, or even a lawyer can be proposed to prepare the request for disability or early retirement as best as possible.
- A wish to change positions, companies, or jobs [40,41]. The wish to change one’s professional life can be explained by a change in life philosophy due to the diagnosis and treatment of cancer, conceptualized as post-traumatic growth [40,41,42]. It will also be a matter of helping the patient to identify new professional opportunities, (i.e., social liberation) before hoping to reach the preparation phase [14]. Contact with a social worker or a service specialized in professional integration could be helpful.

To create a good therapeutic alliance, it is important to respect the patient’s choice and to give her the necessary time for reflection to motivate her to begin the preparation phase, whatever the decision. If necessary, several appointments can be scheduled to support the reflection on the clarification of values and the RTW project. It is important that the main elements of the discussion be recorded and validated by the patient in a follow-up booklet (Table 1). This allows both the patient and the RTW coordinator to report on the evolution of the reflections regarding the values related to the future professional project.

### 4.4. Stage 3—Preparation

When the BCS feels ready to prepare her RTW, the RTW coordinator can offer a tailored intervention [11,12,43]. The objective is twofold: (i) to promote work ability by improving health-related quality of life, according to the BCS’s needs [13]; and (ii) to promote adjustments to the work situation, work organization, and management practices as much as possible [9].

***Promoting work ability by improving health-related quality of life.*** Interventions promoting health-related quality of life are well known (e.g., physical activity, psychological support, peer support, etc.) [26,27]. However, it is more complex to tailor these interventions to the patient’s specific needs. A screening tool, specifically designed to assess the main determinants of RTW, could be proposed and recorded in the follow-up booklet in order to identify the most appropriate intervention (Table 1) [13]. This screening tool will allow a multidisciplinary working group, (i.e., RTW coordinator, healthcare professionals, occupational health physician, specialized nurse, psychologist, ergonomist, physical activity specialist, physiotherapist, social worker) to propose several support options to the BCS [8]. The BCS will choose the one (or those) she is most motivated to carry out, since motivation is an important prognostic factor of adherence to supportive care [44].

***Promoting adjustments to the work situation, work organization, and management practices as much as possible.*** BCSs have no legal obligation to precisely specify the reasons for their sick leave to their employer [30]. It is ethically and legally inappropriate for healthcare professionals to directly contact the manager or employer of an employee who has been diagnosed with cancer without the patient’s consent [45], even to propose tools to support the sustainable RTW. Such contact should be legally established by the BCS herself, or via the occupational health physician with the consent of the patient, while considering medical privacy. The BCS is then free to choose whether or not to pass on the necessary information to her manager. It is therefore possible to remind her that she can pass on the informative website to her manager transmitted during the pre-contemplation phase (Table 1), in which a section is reserved for managers and employers, (e.g., MiLES) [9,28,29]. If accepted, the manager is then free to consult this website. It is also possible to include the contact details of the RTW coordinator, occupational health physician, work psychologists, or ergonomists, in case of additional questions or even more individualized support for the manager.

***Identifying both health- and work-related difficulties (Table 4)***. All the variables are listed in Table 4.

**Figure 1:**
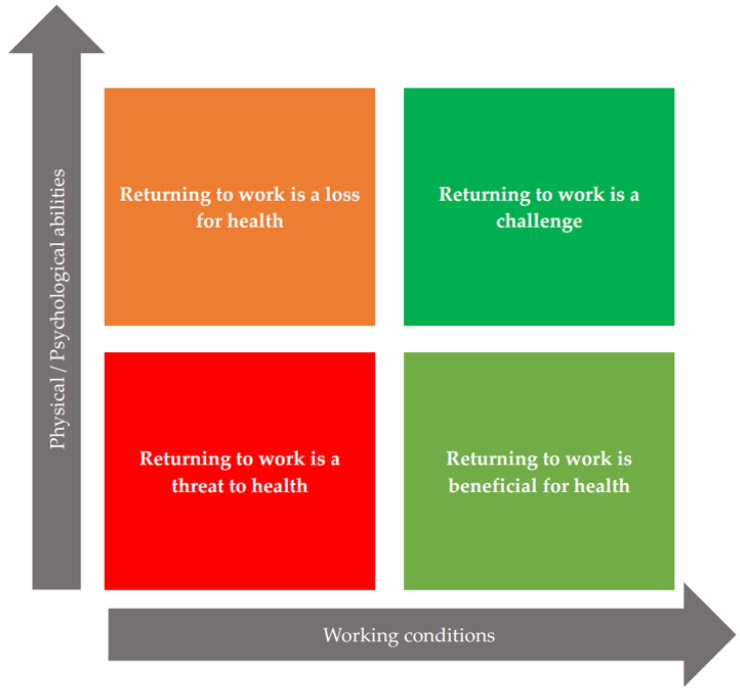
Profiles of patients according to physical/psychological abilities and working conditions.

The relationship between the physical/psychological abilities of the BCSs and the former work situation in terms of initial MM will provide a first assessment of the RTW situation (Figure 1) [13]. Wage loss and quality of the care journey (including the quality of the met supportive care) should complement this assessment as they may be perceived as sources of stress that impact the BCS’ psychological health [46,47]. As each BCS is unique, other elements that are not mentioned in the REWORK-BC model can also be noted in a more qualitative way, (e.g., health problems other than cancer, possible relationship problems within the couple or family, children’s school difficulties, health problems of the spouse, children or any other family member).

Considering individual, social and financial resources will help to nuance the first assessment [13]. The intervention proposal will therefore have to consider whether the BCS feels able to RTW and whether she is well supported. Finally, it is essential to assess individual coping skills and work accommodation options [48]. It should be remembered that some BCSs can be successful in the RTW on their own, against all expectations [13,49].

***Promoting the achievement of the action phase: self-liberation.*** In accordance with the TTM and REWORK-BC models, a tailored intervention will aim to improve work ability and resources so that the BCS’ RTW self-efficacy and coping skills will increase [13,14]. All elements questioned (Table 4) must be re-evaluated as many times as necessary during the preparation phase (time intervals to be determined by the multidisciplinary team and by the BCS herself) and noted in the follow-up booklet (Table 1). This will allow the BCS, and the multidisciplinary team to see the progress. As soon as the RTW is identified as beneficial for health (Figure 1) and the BCS feels able to RTW, the pre-RTW visit can take place and will be facilitated by the elements annotated in the follow-up booklet (Table 1). The occupational health physician can then set a date for the RTW in agreement with the BCS and the employer, and make the final work accommodations necessary for a successful RTW [13,48]. For patients in a situation where the RTW is a threat to their health (Figure 1), and/or who do not wish to return to their former work, supporting intervention towards a change of employment, (e.g., social worker, occupational psychologist, specialized devices) is essential to prepare future job recruitment [14].

### 4.5. Stage 4—Action

At this point, the BCS has returned to work but is likely to be on sick leave several times [50]. It is therefore necessary for the RTW coordinator or the occupational health physician to offer her regular appointments in order to make an overall assessment of her values about work, her work ability, the resources at her disposal, and her coping skills (Table 3 and Table 4) [13]. All these elements must be filled in the follow-up booklet (Table 1). This will allow the necessary adjustments to be made for a sustainable RTW, (i.e., sufficient final MM). In case of sick leave, the follow-up appointments will reassure the BCS by explaining that it is part of the process [14], that there is no right or wrong way to RTW; the important thing being to preserve her health. In some cases, BCSs will start the process again with the goal of changing their professional life.

***Promoting the achievement of the maintenance phase.*** Regular motivational appointments can be organized by the RTW coordinator or the occupational health physician to help the BCS assert herself. They can also provide advice on how to better organize her work, given the long-term side effects (especially physical and cognitive fatigue) [4,34]. It is also preferable to modify the work environment, (e.g., change the layout of the office, have new assignments) [48]. To achieve this, relationships with managers and colleagues must be favorable [6,9]. Managers or employers who have agreed to participate in the program, (e.g., MiLES) may also be offered counseling if the information made available on the website does not seem sufficient (Table 1) [9,28,29].

### 4.6. Stage 5—Maintenance

The maintenance phase is relatively similar to the action phase. Because sick leave is less frequent, the time intervals between interviews are longer. Follow-up appointments are recommended but should be done with the needs and preferences of the BCS. The elements must be noted in the follow-up booklet (Table 1) and transmitted to the occupational physician so that he/she can make progressive readjustments.

### 4.7. Stage 6—Termination of the Process

The process ends when: (i) the BCS has not been off work, due to cancer and its treatments, for more than six months; (ii) the BCS is satisfied with the new sustainable working situation; and (iii) the BCS demonstrates satisfactory health- and work-related quality of life [51].

## 5. Measurements and Assessment Indicators for Each Stage of the Process

Regarding the REWORK-BC model, many psychosocial variables need to be collected throughout the process [4,13]. Some questionnaires are commonly used in oncology or occupation health studies for assessing these psychosocial variables, for example, the EORTC-QLQ-C30, (i.e., quality of life) [52], the Hospital Anxiety and Depression Scale [53], the Multidimensional Fatigue Inventory [54,55], the Post-traumatic Growth Inventory [56,57,58], the Body Image Scale [59], the RTW Self-Efficacy scale [38,60,61], the Cognitive Symptom Checklist-Work [62,63], the Job Content Questionnaire [64], or the Copenhagen Psychosocial Questionnaire [65,66]. However, filling out numerous questionnaires, although submitted sequentially, may be cognitively challenging for BCSs who have potentially been treated with chemotherapy [67]. In this sense, we encourage the use of short forms of questionnaires, and future research to propose more flexible assessment strategies, facilitating the collection of information from patients and allowing time for discussion from a clinical perspective.

In addition, some indicators of the progress of the BCS in the different stages of change of the TTM [14] could also be proposed as follows:

- *Stage 2—Contemplation:* the BCS mentions the issue of the RTW and indicates that the documents and websites made available to her were instrumental in getting her to talk about it. The BCS also provides the necessary information to her manager or employer.
- *Stage 3—Preparation:* the BCS indicates that she is clear with her values and testifies to a good therapeutic alliance with the RTW coordinator. Contact is established with the manager.
- *Stage 4—Action:* the BCS has a greater RTW self-efficacy than before the preparation, her health-related quality of life is favorable, and the manager or employer is prepared to welcome her under good working conditions.
- *Stage 5—Maintenance:* the BCS shows a good health- and work-related quality of life after RTW. Sick leave due to cancer and its treatment is less frequent.
- *Stage 6—Termination of the process:* the BCS shows a good health- and work-related quality of life. There is no more sick leave due to cancer or its treatment.

## 6. Conclusions

The proposed clinical framework is based on: (i) a relevant theoretical model frequently used for promoting the health-related quality of life of cancer patients and survivors, (i.e., TTM); (ii) a model specific to the RTW process of BCSs based on expert consensus, (i.e., REWORK-BC model); and (iii) the scientific literature focusing on the RTW of BCSs. It offers all healthcare and occupational health professionals a practical guide to better understand the RTW process of BCSs. By adapting the determinants related to the medical treatments and some specific side effects, we argue that this clinical framework could be extrapolated to other tumor types. The flexibility of the framework and the implementation of simple tools, either already available or to be developed in future studies, make it adaptable to various health systems around the world. While this clinical framework is partly based on a model resulting from a French consensus, it aims to be a usable tool for any hospital or cancer care center wishing to implement a patient-centered intervention to promote the RTW of BCSs, regardless of the country.

## Figures and Tables

**Table 1:** Functionality of simple tools.

TTM Phase	Process to Achieve	Simple Tool	Functionality
*1. Precontemplation*	Consciousness-raising	Poster in medical offices	Encourage the BCS to talk about her RTW with her referring physician as soon as possible.
	Dramatic relief	Informative booklet or leaflet [33]	Convey essential information about the RTW process.
	Environmental re-evaluation	Website (e.g., MiLES) [9,28,29]	Share testimonials from patients and managers who have been through the same process. Inform the manager, as soon as possible, about good managerial practices (only if the patient agrees to pass them on). Provide an opportunity for the manager to seek assistance in promoting a sustainable RTW (only if the patient agrees to pass them on).
*2. Contemplation*	Self-re-evaluation	Follow-up booklet	Keep a written record of the values and be aware of the evolution throughout the reflection (Table 3). Encourage shared representation with the RTW coordinator.
*3. Preparation*	Self-liberation	Screening tool [13]	Identify the most appropriate procedure for the BCS (Table 4).
		Follow-up booklet	Keep a written record; be aware of the evolution throughout the tailored intervention. Encourage shared representation with the multidisciplinary team involved in the tailored intervention. Prepare the pre-RTW visit with the occupational health physician.
		Website (e.g., MiLES) [9,28,29]	Inform the manager of good managerial practices (only if the patient agrees to pass them on). Provide an opportunity for the manager to seek assistance in promoting a sustainable RTW (only if the BCS agrees to pass them on).
*4. Action*	Counterconditioning Helping relationships Reinforcement management Stimulus control	Screening tool	Carry out the necessary reassessments (Tables 3 and 4)
		Follow-up booklet	Keep a written record; be aware of the evolution throughout the tailored intervention. Encourage shared representation with the multidisciplinary team involved in the tailored intervention. Prepare the appointment with the occupational health physician if necessary.
		Website (e.g., MiLES) [9,28,29]	Inform the manager of good managerial practices (only if the patient agrees to pass them on). Provide an opportunity for the manager to seek assistance in promoting a sustainable RTW (only if the BCS agrees to pass them on).
*5. Maintenance*		Screening tool	Carry out the necessary reassessments (Tables 3 and 4)
		Follow-up booklet	Keep a written record; be aware of the evolution throughout the tailored intervention. Encourage shared representation with the multidisciplinary team involved in the tailored intervention. Prepare the appointment with the occupational health physician if necessary.
		Website (e.g., MiLES) [9,28,29]	Inform the manager of good managerial practices (only if the patient agrees to pass them on). Provide an opportunity for the manager to seek assistance in promoting a sustainable RTW (only if the BCS agrees to pass them on).

Notes. MiLES: the Missing Link: optimizing RTW of Employees diagnosed with cancer, by Supporting employers. TTM: Transtheoretical Model of Change.

**Table 2:** Reminder of the variables to be recorded in the medical file.

**Socio-demographic**	☐ Age	☐ Education
	☐ Ethnicity	☐ Place of residence
	☐ Dependent children	☐ Social precariousness
**Professional**	☐ Socio-professional category	☐ Professional status
	☐ Company size	☐ Type of contract
	☐ Seniority in the company	☐ Hierarchical position
**Financial**	☐ Income	☐ Main family breadwinner
**Medical**	☐ Cancer stage	☐ First cancer diagnosis or more
	☐ Type of surgery	☐ Chemotherapy
	☐ Radiation therapy	☐ Hormone therapy
	☐ Immunotherapy	☐ Targeted therapy (e.g., Trastuzumab)

**Table 3:** Discussion points for value clarification.

☐ Intention to RTW
☐ Meaning of work
☐ Work attachment
☐ Sense of professional usefulness
☐ Post-traumatic growth
☐ Wage losses and/or financial consequences
☐ RTW self-efficacy (if necessary)

**Table 4:** Reminder of the variables to be recorded at the preparation phase.

**Physical abilities**	☐ Overall health status
	☐ Physical fatigue
	☐ Disability due to BC (physical sequelae, pain, restricted arm movement)
**Psychological abilities**	☐ Emotional distress (anxiety, depression)
	☐ Emotional fatigue
	☐ Cognitive fatigue
	☐ Body image
**Working conditions**	☐ Work-related stressors (physical, psychological, organizational)
**Others**	☐ Wage loss
	☐ Quality of the care journey (and the met supportive care)
	☐ Any other elements to be noted in a qualitative way
**Resources**	☐ RTW self-efficacy
	☐ Perceived social support (colleagues, managers, medical staff, family, friends)
	☐ Recognition by colleagues and/or managers
**Adjustments**	☐ Coping (benefit finding, problem-focused, emotion-focused)
	☐ Work accommodations (working time, workstation, professional duties)

## Data Availability

Not applicable.

## References

[1] Bray F., Ferlay J., Soerjomataram I., Siegel R.L., Torre L.A., Jemal A. (2018). Global Cancer Statistics 2018: GLOBOCAN Estimates of Incidence and Mortality Worldwide for 36 Cancers in 185 Countries. CA Cancer J. Clin..

[2] Shapiro C.L. (2018). Cancer Survivorship. N. Engl. J. Med..

[3] Wang L., Hong B.Y., Kennedy S.A., Chang Y., Hong C.J., Craigie S., Kwon H.Y., Romerosa B., Couban R.J., Reid S. (2018). Predictors of Unemployment After Breast Cancer Surgery: A Systematic Review and Meta-Analysis of Observational Studies. J. Clin. Oncol..

[4] Porro B., Bertin M., Bonnaud Antignac A., Petit A., Cousson-Gélie F., Roquelaure Y. (2019). Assessment of Psychosocial Dimensions of Return to Work after a Cancer Diagnosis: Current Perspectives and Future Opportunities. Psychooncology.

[5] Brusletto B., Torp S., Ihlebæk C.M., Vinje H.F. (2018). A Five-Phase Process Model Describing the Return to Sustainable Work of Persons Who Survived Cancer: A Qualitative Study. Eur. J. Oncol. Nurs..

[6] Greidanus M.A., de Boer A.G.E.M., de Rijk A.E., Tiedtke C.M., Dierckx de Casterlé B., Frings-Dresen M.H.W., Tamminga S.J. (2018). Perceived Employer-Related Barriers and Facilitators for Work Participation of Cancer Survivors: A Systematic Review of Employers’ and Survivors’ Perspectives. Psychooncology.

[7] Feuerstein M., Todd B.L., Moskowitz M.C., Bruns G.L., Stoler M.R., Nassif T., Yu X. (2010). Work in Cancer Survivors: A Model for Practice and Research. J. Cancer Surviv. Res. Pract..

[8] Cohen M., Yagil D., Carel R. (2021). A Multidisciplinary Working Model for Promoting Return to Work of Cancer Survivors. Support. Care Cancer.

[9] Greidanus M.A., Tamminga S.J., de Rijk A.E., Frings-Dresen M.H.W., de Boer A.G.E.M. (2019). What Employer Actions Are Considered Most Important for the Return to Work of Employees with Cancer? A Delphi Study Among Employees and Employers. J. Occup. Rehabil..

[10] Lamort-Bouché M., Péron J., Broc G., Kochan A., Jordan C., Letrilliart L., Fervers B., Fassier J.-B. (2020). FASTRACS Group Breast Cancer Specialists’ Perspective on Their Role in Their Patients’ Return to Work: A Qualitative Study. Scand. J. Work. Environ. Health.

[11] Lamore K., Dubois T., Rothe U., Leonardi M., Girard I., Manuwald U., Nazarov S., Silvaggi F., Guastafierro E., Scaratti C. (2019). Return to Work Interventions for Cancer Survivors: A Systematic Review and a Methodological Critique. Int. J. Environ. Res. Public. Health.

[12] Caron M., Durand M.-J., Tremblay D. (2017). Interventions to support the return-to-work process after cancer: A literature review. Sante Publique Vandoeuvre-Nancy Fr..

[13] Porro B., Durand M.-J., Petit A., Bertin M., Roquelaure Y. (2021). Return to Work of Breast Cancer Survivors: Toward an Integrative and Transactional Conceptual Model. J. Cancer Surviv..

[14] Prochaska J.O., Redding C.A., Evers K.E. (2015). The Transtheoretical Model and Stages of Change. Health Behavior: Theory, Research, and Practice.

[15] Duijts S.F.A., Bleiker E.M.A., Paalman C.H., Beek A.J. (2017). van der A Behavioural Approach in the Development of Work-Related Interventions for Cancer Survivors: An Exploratory Review. Eur. J. Cancer Care.

[16] Zegers A.D., Coenen P., Bültmann U., van Hummel R., van der Beek A.J., Duijts S.F.A. (2022). Tailoring Work Participation Support for Cancer Survivors Using the Stages of Change: Perspectives of (Health Care) Professionals and Survivors. J. Cancer Surviv..

[17] Prochaska J.O., Velicer W.F., DiClemente C.C., Fava J. (1988). Measuring Processes of Change: Applications to the Cessation of Smoking. J. Consult. Clin. Psychol..

[18] Prochaska J.O., DiClemente C.C., Shiffman S., Wills T. (1985). Common Processes of Change for Smoking, Weight Control, and Psychological Distress. Coping and Substance Abuse.

[19] Prochaska J.O., DiClemente C.C. (1982). Transtheoretical Therapy: Toward a More Integrative Model of Change. Psychother. Theory Res. Pract..

[20] Durand M.-J., Vézina N., Richard M.-C., Schultz I.Z., Gatchel R.J. (2016). Concept of Margin of Manoeuvre in Return to Work. Handbook of Return to Work: From Research to Practice.

[21] Durand M.J., Vézina N., Baril R., Loisel P., Richard M.C., Ngomo S. (2009). Margin of Manoeuvre Indicators in the Workplace During the Rehabilitation Process: A Qualitative Analysis. J. Occup. Rehabil..

[22] Christy S.M., Mosher C.E., Sloane R., Snyder D.C., Lobach D.F., Demark-Wahnefried W. (2011). Long-Term Dietary Outcomes of the FRESH START Intervention for Breast and Prostate Cancer Survivors. J. Am. Diet. Assoc..

[23] Demark-Wahnefried W., Morey M.C., Sloane R., Snyder D.C., Miller P.E., Hartman T.J., Cohen H.J. (2012). Reach out to Enhance Wellness Home-Based Diet-Exercise Intervention Promotes Reproducible and Sustainable Long-Term Improvements in Health Behaviors, Body Weight, and Physical Functioning in Older, Overweight/Obese Cancer Survivors. J. Clin. Oncol. Off. J. Am. Soc. Clin. Oncol..

[24] Emmons K.M., Puleo E., Park E., Gritz E.R., Butterfield R.M., Weeks J.C., Mertens A., Li F.P. (2005). Peer-Delivered Smoking Counseling for Childhood Cancer Survivors Increases Rate of Cessation: The Partnership for Health Study. J. Clin. Oncol. Off. J. Am. Soc. Clin. Oncol..

[25] Hashemzadeh M., Rahimi A., Zare-Farashbandi F., Alavi-Naeini A.M., Daei A. (2019). Transtheoretical Model of Health Behavioral Change: A Systematic Review. Iran. J. Nurs. Midwifery Res..

[26] Duncan M., Moschopoulou E., Herrington E., Deane J., Roylance R., Jones L., Bourke L., Morgan A., Chalder T., Thaha M.A. (2017). Review of Systematic Reviews of Non-Pharmacological Interventions to Improve Quality of Life in Cancer Survivors. BMJ Open.

[27] Zeng Y., Dong J., Huang M., Zhang J., Zhang X., Xie M., Wefel J.S. (2020). Nonpharmacological Interventions for Cancer-Related Cognitive Impairment in Adult Cancer Patients: A Network Meta-Analysis. Int. J. Nurs. Stud..

[28] Greidanus M.A., de Rijk A.E., Frings-Dresen M.H.W., Tiedtke C.M., Brouwers S., de Boer A.G.E.M., Tamminga S.J. (2020). The Use and Perceived Usefulness of an Online Toolbox Targeted at Employers (MiLES Intervention) for Enhancing Successful Return to Work of Cancer Survivors. J. Occup. Rehabil..

[29] Greidanus M.A., de Boer A.G.E.M., Tiedtke C.M., Frings-Dresen M.H.W., de Rijk A.E., Tamminga S.J. (2020). Supporting Employers to Enhance the Return to Work of Cancer Survivors: Development of a Web-Based Intervention (MiLES Intervention). J. Cancer Surviv..

[30] Tamminga S.J., Braspenning A.M., Haste A., Sharp L., Frings-Dresen M.H.W., de Boer A.G.E.M. (2018). Barriers to and Facilitators of Implementing Programs for Return to Work (RTW) of Cancer Survivors in Four European Countries: A Qualitative Study. J. Occup. Rehabil..

[31] de Rijk A., Amir Z., Cohen M., Furlan T., Godderis L., Knezevic B., Miglioretti M., Munir F., Popa A.E., Sedlakova M. (2020). The Challenge of Return to Work in Workers with Cancer: Employer Priorities despite Variation in Social Policies Related to Work and Health. J. Cancer Surviv..

[32] Loisel P., Buchbinder R., Hazard R., Keller R., Scheel I., van Tulder M., Webster B. (2005). Prevention of Work Disability Due to Musculoskeletal Disorders: The Challenge of Implementing Evidence. J. Occup. Rehabil..

[33] Nieuwenhuijsen K., Bos-Ransdorp B., Uitterhoeve L.L.J., Sprangers M.A.G., Verbeek J.H.A.M. (2006). Enhanced Provider Communication and Patient Education Regarding Return to Work in Cancer Survivors Following Curative Treatment: A Pilot Study. J. Occup. Rehabil..

[34] Porro B., Michel A., Zinzindohoué C., Bertrand P., Monrigal E., Trentini F., Baussard L., Cousson-Gélie F. (2019). Quality of Life, Fatigue and Changes Therein as Predictors of Return to Work during Breast Cancer Treatment. Scand. J. Caring Sci..

[35] Duijts S.F.A., van Egmond M.P., Spelten E., van Muijen P., Anema J.R., van der Beek A.J. (2014). Physical and Psychosocial Problems in Cancer Survivors beyond Return to Work: A Systematic Review. Psychooncology.

[36] Tan H.S.K., Yeo D.S.C., Giam J.Y.T., Cheong F.W.F., Chan K.F. (2016). A Randomized Controlled Trial of a Return-to-Work Coordinator Model of Care in a General Hospital to Facilitate Return to Work of Injured Workers. Work Read. Mass.

[37] Durand M.-J., Nastasia I., Coutu M.-F., Bernier M. (2017). Practices of Return-to-Work Coordinators Working in Large Organizations. J. Occup. Rehabil..

[38] Porro B., de Boer A.G.E.M., Frings-Dresen M.H.W., Roquelaure Y. (2020). Self-Efficacy and Return to Work in Cancer Survivors: Current Knowledge and Future Prospects. Eur. J. Cancer Care.

[39] Lindbohm M.-L., Kuosma E., Taskila T., Hietanen P., Carlsen K., Gudbergsson S., Gunnarsdottir H. (2014). Early Retirement and Non-Employment after Breast Cancer: Non-Employment after Breast Cancer. Psychooncology.

[40] Nilsson M.I., Olsson M., Wennman-Larsen A., Petersson L.-M., Alexanderson K. (2013). Women’s Reflections and Actions Regarding Working after Breast Cancer Surgery—A Focus Group Study: Reflections and Actions Regarding RTW after Breast Cancer. Psychooncology.

[41] Tiedtke C., Dierckx de Casterlé B., de Rijk A., Christiaens M.-R., Donceel P. (2011). Breast Cancer Treatment and Work Disability: Patient Perspectives. Breast Edinb. Scotl..

[42] Tedeschi R.G., Calhoun L.G. (2004). Posttraumatic Growth: Conceptual Foundations and Empirical Evidence. Psychol. Inq..

[43] Bilodeau K., Tremblay D., Durand M.-J. (2017). Exploration of Return-to-Work Interventions for Breast Cancer Patients: A Scoping Review. Support. Care Cancer.

[44] Pudkasam S., Feehan J., Talevski J., Vingrys K., Polman R., Chinlumprasert N., Stojanovska L., Apostolopoulos V. (2021). Motivational Strategies to Improve Adherence to Physical Activity in Breast Cancer Survivors: A Systematic Review and Meta-Analysis. Maturitas.

[45] Greidanus M.A., de Rijk A.E., de Boer A.G.E.M., Bos M.E.M.M., Plaisier P.W., Smeenk R.M., Frings-Dresen M.H.W., Tamminga S.J. (2021). A Randomised Feasibility Trial of an Employer-Based Intervention for Enhancing Successful Return to Work of Cancer Survivors (MiLES Intervention). BMC Public Health.

[46] Martínez Arroyo O., Andreu Vaíllo Y., Martínez López P., Galdón Garrido M.J. (2019). Emotional Distress and Unmet Supportive Care Needs in Survivors of Breast Cancer beyond the End of Primary Treatment. Support. Care Cancer.

[47] Lauzier S., Lévesque P., Mondor M., Drolet M., Coyle D., Brisson J., Mâsse B., Provencher L., Robidoux A., Maunsell E. (2013). Out-of-Pocket Costs in the Year After Early Breast Cancer Among Canadian Women and Spouses. J. Natl. Cancer Inst..

[48] Alleaume C., Paraponaris A., Bendiane M.-K., Peretti-Watel P., Bouhnik A.-D. (2020). The Positive Effect of Workplace Accommodations on the Continued Employment of Cancer Survivors Five Years after Diagnosis. Support. Care Cancer Off. J. Multinatl. Assoc. Support. Care Cancer.

[49] van Muijen P., Schellart A.J.M., Duijts S.F.A., Beek A.J. (2019). van der The Mediating Role of Coping between Self-Reported Health Complaints and Functional Limitations, Self-Assessed Work Ability and Work Status of Long-Term Sick-Listed Cancer Survivors. Eur. J. Cancer Care.

[50] Chen L., Alexanderson K.A.E. (2020). Trajectories of Sickness Absence and Disability Pension in the 2 Years before and 3 Years after Breast Cancer Diagnosis: A Swedish Longitudinal Population-based Cohort Study. Cancer.

[51] Greidanus M.A., de Boer A.G.E.M., de Rijk A.E., Brouwers S., de Reijke T.M., Kersten M.J., Klinkenbijl J.H.G., Lalisang R.I., Lindeboom R., Zondervan P.J. (2020). The Successful Return-To-Work Questionnaire for Cancer Survivors (I-RTW_CS): Development, Validity and Reproducibility. Patient-Patient-Cent. Outcomes Res..

[52] Aaronson N.K., Ahmedzai S., Bergman B., Bullinger M., Cull A., Duez N.J., Filiberti A., Flechtner H., Fleishman S.B., de Haes J.C. (1993). The European Organization for Research and Treatment of Cancer QLQ-C30: A Quality-of-Life Instrument for Use in International Clinical Trials in Oncology. J. Natl. Cancer Inst..

[53] Zigmond A.S., Snaith R.P. (1983). The Hospital Anxiety and Depression Scale. Acta Psychiatr. Scand..

[54] Smets E.M., Garssen B., Bonke B., de Haes J.C.J.M. (1995). The Multidimensional Fatigue Inventory (MFI). Psychometric Qualities of an Instrument to Assess Fatigue. J. Psychosom. Res..

[55] Baussard L., Carayol M., Porro B., Baguet F., Cousson-Gelie F. (2018). Fatigue in Cancer Patients: Development and Validation of a Short Form of the Multidimensional Fatigue Inventory (MFI-10). Eur. J. Oncol. Nurs..

[56] Brunet J., McDonough M.H., Hadd V., Crocker P.R.E., Sabiston C.M. (2010). The Posttraumatic Growth Inventory: An Examination of the Factor Structure and Invariance among Breast Cancer Survivors. Psychooncology.

[57] Tedeschi R.G., Calhoun L.G. (1996). The Posttraumatic Growth Inventory: Measuring the Positive Legacy of Trauma. J. Trauma. Stress.

[58] Cann A., Calhoun L.G., Tedeschi R.G., Taku K., Vishnevsky T., Triplett K.N., Danhauer S.C. (2010). A Short Form of the Posttraumatic Growth Inventory. Anxiety Stress Coping.

[59] Hopwood P., Fletcher I., Lee A., Al Ghazal S. (2001). A Body Image Scale for Use with Cancer Patients. Eur. J. Cancer Oxf. Engl. 1990.

[60] Lagerveld S.E., Blonk R.W.B., Brenninkmeijer V., Schaufeli W.B. (2010). Return to Work among Employees with Mental Health Problems: Development and Validation of a Self-Efficacy Questionnaire. Work Stress.

[61] Porro B., Petit A., Bourbouloux E., Colombat P., Le-Blanc Onfroy M., Fassier J.-B., Roquelaure Y. (2021). French translation and adaptation of the “Return to Work Self-Efficacy’ Scale-11 items” in patients diagnosed with a cancer. Bull. Cancer.

[62] Ottati A., Feuerstein M. (2013). Brief Self-Report Measure of Work-Related Cognitive Limitations in Breast Cancer Survivors. J. Cancer Surviv. Res. Pract..

[63] Dorland H.F., Abma F.I., Roelen C.A.M., Smink A., Feuerstein M., Amick B.C., Ranchor A.V., Bültmann U. (2016). The Cognitive Symptom Checklist-Work in Cancer Patients Is Related with Work Functioning, Fatigue and Depressive Symptoms: A Validation Study. J. Cancer Surviv. Res. Pract..

[64] Karasek R., Brisson C., Kawakami N., Houtman I., Bongers P., Amick B. (1998). The Job Content Questionnaire (JCQ): An Instrument for Internationally Comparative Assessments of Psychosocial Job Characteristics. J. Occup. Health Psychol..

[65] Burr H., Berthelsen H., Moncada S., Nübling M., Dupret E., Demiral Y., Oudyk J., Kristensen T.S., Llorens C., Navarro A. (2019). The Third Version of the Copenhagen Psychosocial Questionnaire. Saf. Health Work.

[66] Kristensen T.S., Hannerz H., Høgh A., Borg V. (2005). The Copenhagen Psychosocial Questionnaire—A Tool for the Assessment and Improvement of the Psychosocial Work Environment. Scand. J. Work. Environ. Health.

[67] Von Ah D., Storey S., Tallman E., Nielsen A., Johns S., Pressler S. (2016). Cancer, Cognitive Impairment, and Work-Related Outcomes: An Integrative Review. Oncol. Nurs. Forum.

